# Whole brain functional recordings at cellular resolution in zebrafish larvae with 3D scanning multiphoton microscopy

**DOI:** 10.1038/s41598-021-90335-y

**Published:** 2021-05-26

**Authors:** Matteo Bruzzone, Enrico Chiarello, Marco Albanesi, Maria Elena Miletto Petrazzini, Aram Megighian, Claudia Lodovichi, Marco dal Maschio

**Affiliations:** 1 Department of Biomedical Sciences, University of Padua, via U. Bassi 58, Padua, Italy; 2 Padua Neuroscience Center - PNC, University of Padua, via Orus 2B, Padua, Italy; 3 Veneto Institute of Molecular Medicine, VIMM, via Orus 2, Padua, Italy; 4 Institute of Neuroscience, CNR-IN, Padua, Italy

**Keywords:** Neuroscience, Neural circuits, Optics and photonics, Optical techniques, Imaging and sensing

## Abstract

Optical recordings of neuronal activity at cellular resolution represent an invaluable tool to investigate brain mechanisms. Zebrafish larvae is one of the few model organisms where, using fluorescence-based reporters of the cell activity, it is possible to optically reconstruct the neuronal dynamics across the whole brain. Typically, leveraging the reduced light scattering, methods like lightsheet, structured illumination, and light-field microscopy use spatially extended excitation profiles to detect in parallel activity signals from multiple cells. Here, we present an alternative design for whole brain imaging based on sequential 3D point-scanning excitation. Our approach relies on a multiphoton microscope integrating an electrically tunable lens. We first apply our approach, adopting the GCaMP6s activity reporter, to detect functional responses from retinal ganglion cells (RGC) arborization fields at different depths within the zebrafish larva midbrain. Then, in larvae expressing a nuclear localized GCaMP6s, we recorded whole brain activity with cellular resolution. Adopting a semi-automatic cell segmentation, this allowed reconstructing the activity from up to 52,000 individual neurons across the brain. In conclusion, this design can easily retrofit existing imaging systems and represents a compact, versatile and reliable tool to investigate neuronal activity across the larva brain at high resolution.

## Introduction

Understanding how patterns of neuronal activity across the brain support the processing of sensory information and the selection of an appropriate motor program is an intriguing task. Nowadays, only a limited number of model organisms allow an investigation of the underlying circuit mechanisms at the whole brain scale and with cellular resolution^1^. Among others, zebrafish larvae are transparent and have a brain with suitable dimensions either in terms of accessible volume—about 400 × 800 × 250 μm^3^ (W × L × H)—and in the number of neuronal units—estimated between 80,000 and 100,000. Moreover, at the early developmental stages, larvae can already reliably perform a wide range of spontaneous and sensory-evoked behavioral maneuvers, making this model an ideal system to explore the underlying circuit mechanisms^2^. Observation of neuronal activity of large populations across the brain of this model organism has been taking advantage of two fundamental elements. On one side, we have protein engineering. In the last 10 years, the development and the continuous refinement of genetically encoded fluorescent reporters have enabled high sensitivity recordings of physiological events like calcium increases, changes in the transmembrane voltage and release of neurotransmitters. Along with this, on the other side, novel optical technologies and imaging methods have been refined and applied. These allowed to extend the sampling capabilities, in terms of either data bandwidth or sensitivity, or investigation volume^3,4^.

Imaging whole brain activity in zebrafish larvae is currently possible with a few approaches: Selective Plane Illumination Microscopy (SPIM)^5,6^, Structured Illumination Microscopy (SIM)^7^ and Light Field Microscopy (LFM)^8,9^. All these rely on the illumination of the sample with spatially extended illumination profiles, that can be either thin sheets of light, or a series of grating, or a bulk diffuse illumination. Because of the reduced light scattering of the zebrafish larva and the acceptable preservation of the ballistic component of the photons emitted, all these techniques adopt parallel detectors like CMOS or CCD^5^ to sample the activity of many cells within the field of view during the same exposure. In these scenarios, volumetric information is typically reconstructed either by shifting the illumination field in coordination with the detection objective as in SPIM and SIM, or via image deconvolution algorithms for LFM. In the most common design, SPIM is obtained by rapidly scanning horizontally a thin laser beam with a galvo mirror^10^; a piezo actuator moves the objective and the imaged plane at different levels, which are sequentially illuminated by means of a second galvo mirror that steers the light sheet along the z axis of the sample. Even though this approach is very effective, continuously moving a 200–300 g objective at 1–2 Hz at a distance of 1–2 mm from the sample, easily represents a perturbation in the acquisition, either in terms of background noise or as acoustic vibrations mechanically transferred to the sample. For this purpose, several techniques, not limited to lightsheet applications, have been developed to quickly access the depth dimension avoiding the movement of the objective^4^. This is typically achieved by remotely modulating the beam convergence properties upstream of the objective using active optical components. Examples of such solutions are Spatial Light Modulators (SLMs)^11–13^, Deformable Mirrors (DMs)^14^, Acousto-Optic Deflectors (AODs)^15–17^, lenses with a programmable focal length^18–22^ or objective-based z-scanning secondary arms^23–25^. Among these, active lenses are the simplest configuration to integrate and control. Electrically Tunable Lenses (ETLs) are lenses composed of a liquid volume enclosed between elastic polymer membranes. An electromagnetic coil driven by an electric current exerts pressure on the liquid, increasing the curvature of the membrane and thus the focal power of the lens. This allows to move the focal point of the system along its optical axis while keeping the objective still. These remote focusing approaches are significantly extending the possibility to apply optical methods in the investigation of the brain dynamics.

Here, we report on a novel hardware design for recording neuronal activity across the three dimensions of the brain of a restrained larva. With respect to the current techniques, our design is based on a traditional multiphoton microscope equipped with an 8-kHz resonant scanner and integrating, upstream of it, a remote focusing system based on an electrically tunable lens. This layout enables functional recording from a volume of 800 × 400 × 180 μm^3^ with cellular resolution on about 80% of this volume. We first report on the implementation of the design and on the characterization of the optical figures of the system. Then, using a genetically encoded fluorescence-based reporter GCaMP6s, we present a series of functional recordings from the RGC axonal projections of 5 days post fertilization (dpf) zebrafish larvae during the presentation of visual stimuli. Finally, in combination with a refined analysis pipeline, we show that with this configuration it is possible to reconstruct at once the activity from 45,000 to 52,000 cells within the brain of a larva.

## Results

### Configuration for 3D scanning multiphoton microscopy

**Figure 1:**
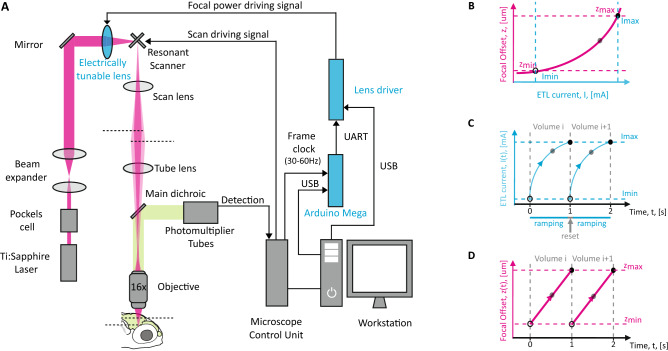
The layout of the optical configuration and the properties of the ETL. (**A**) Implementation of the electrically tunable lens along the optical path, the lens driver and the microcontroller (blue components). The image was generated using Adobe Illustrator CS2 (https://adobe.com/products/illustrator). (**B**) The ETL Look-Up-Table (LUT). Relationship between the input current to the ETL (blue axis) and the change in the focus resulting at the level of the sample (magenta axis). (**C**) The ETL input current works in a quasi-sawtooth pattern. The pattern is composed of two phases. A ramping one, where the input current to the ETL is continuously increased according to the LUT; and a reset one, where the input current is reset to 0 mA. The cycle occurs every 30 frames. (**D**) At the level of the sample, this pattern is translated in a continuous and linear change of the focus.

Aiming at reconstructing neuronal dynamics at cellular resolution in 3D, we designed a raster scanning configuration integrating an electrically tunable lens on a commercial multiphoton microscope equipped with an 8-kHz resonant scanner (Fig. 1A). In our design, the ETL is placed upstream and in close proximity to the galvo mirrors, in a position where the imaging beam fits without overfilling the optical window of the lens^26^. The ETL works in continuous z-scanning mode with the focal position linearly moving during the acquisition of the volume (ramping phase) followed by a quick return to the starting position (reset phase). At this purpose, a quasi-sawtooth waveform is synthesized to control the ETL driving current (Fig. 1C, Cerulean line), according to a pre-calibrated look-up table (LUT, Fig. 1B, Supplementary Fig. S1) based on the volume scanning settings. This LUT links the ETL driving current to the effective position of the focus, allowing to control the excitation spot in the defined z-range (*zmin, zmax*) with an ETL-current value in the corresponding range (*Imin, Imax*) (Fig. 1D, magenta line). In this kind of optical layout, it is essential to maintain a tight synchronization between the lateral (XY) and longitudinal (Z) scanning to avoid undesired artifacts. In order to minimize possible drifts and jitters in the image acquisition, we designed a simple electronic circuit based on an Arduino Mega microcontroller. Briefly, the frame clock generated by the microscope control unit (Frame Sync, see “Methods” section) is used as a clock on a digital incrementing counter implemented on the microcontroller board which controls the ETL driver. The microcontroller is programmed to start the generation of the current ramp according to the measured LUT every time a defined number of frames are reached by the counter. Control of the ETL driving current relies on sending serial commands from the Arduino Mega to the ETL driver unit, encoding current values I(t), corresponding to the expected time t and position z, and calculated on the basis of the LUT.

### Optical figures of the 3D scanning configuration

**Figure 2:**
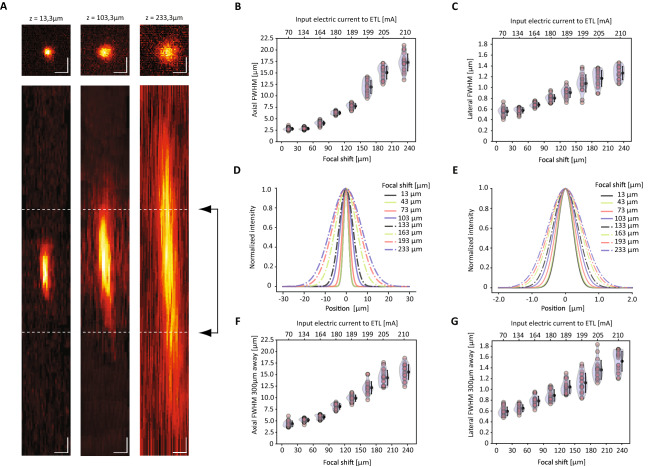
Optical performances of the system. (**A**) Average intensity projections (upper row with top-view XY, lower row with side view ZX) of the point spread function (PSF) measured at 920 nm on 0.2 μm fluorescent beads at three different settings of the ETL tuning range, z = 13.3 μm, z = 103.3 μm and z = 233.3 μm. Highlighted with dashed lines is the typical cell size (6 μm). Scale bar = 1 μm. (**B,C**) Axial and lateral FWHM of the PSF as function of the ETL defocus and of the corresponding ETL driving current, for beads measured within 50 μm from the center of the FOV. (**D,E**) Axial and lateral intensity profile of the PSF for beads measured within 50 μm from the center, corresponding to eight different settings of the ETL. (**F,G**) Axial and lateral FWHM of the PSF as function of the ETL defocus and of the corresponding ETL driving current, for beads measured at 300 μm from the center of the FOV.

Integrating components for the remote control of the focus, like ETLs, generally comes with position-dependent modifications of the optical properties, especially impacting on the effective spatial resolution and the size of the field of view (FOV). To evaluate these aspects, we first assessed the resolution of the system across the volume of interest quantifying the lateral and axial extension of the Point Spread Function (PSF, full width half maximum along the x–y and z directions, FWHM_x,y_ and FWHM_z_, Fig. 2). For eight different positions set with the remote focus in the z-range between 0 and 233 μm, we acquired, mechanically moving the objective with respect to the sample, a corresponding series of z-stacks of fluorescent polystyrene beads with a nominal diameter of 0.2 μm. For the 16× objective used for this study (Nikon CFI75, 16× Water Immersion, NA 0.8, WD 3 mm), data showed a progressive increase in the lateral and axial size of the PSF with the amount of defocus introduced using the ETL. For beads located at the center of the FOV, in the considered z-range, the FWHM_x,y_ increased from 0.54 μm ± 0.08 μm (mean ± standard deviation) to 1.25 μm ± 0.12 μm and the FWHM_z_ from 2.82 μm ± 0.30 μm to 17.28 μm ± 1.88 μm (Fig. 2A–E). Given non-uniform diffraction efficiency of the optical elements, optical performances can easily undergo progressive degradation depending on the radial distance from the center of the field of view. At 300 μm from the FOV origin, for the same z-range between 0 and 233 μm, the PSF presented comparable trends: FWHM_x,y_ increases from 0.59 μm ± 0.08 μm (mean ± standard deviation) to 1.52 μm ± 0.18 μm and the FWHM_z_ from 4.4 μm ± 0.61 μm to 15.5 μm ± 1.61 μm (Fig. 2F–G). As for the deformation of the FOV, from the plane corresponding to the lowest z-level (0 μm) to the plane with the maximal defocus set with the ETL (233 μm), the change in the effective size of the imaged FOV patch is minimal and corresponds to about 3% with respect to the area addressed with the ETL set at the middle of its z-range (Supplementary Fig. S2).

### Multiplane imaging of the retinal ganglion cells (RGC) arborization fields

We initially tested our design by recording neuronal activity from zebrafish larvae expressing the genetically encoded calcium indicator GCaMP6s in a population of retinal ganglion cells (line Ath5:GCaMP6s). The adopted zebrafish line presents a dense labelling of the neuropil regions corresponding to the arborization fields (AFs) of the RGC axonal projections, spanning about 170 μm along the dorsoventral axis of the zebrafish larva brain. The imaging covered a volume of 100 × 100 × 55 μm^3^ with 12 planes equally spaced containing RGC axonal projections (W × L × H, 512 × 512 × 12 voxels) at an acquisition rate of 1 volume per second.

**Figure 3:**
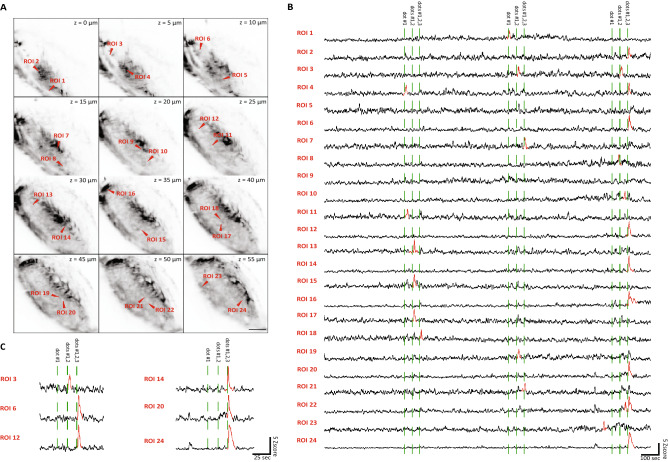
Multiplane calcium imaging of the RGC arborization fields. (**A**) Average intensity projections of the planes acquired across the RGC arborization fields. Red arrowheads indicate the regions of interest (ROIs) corresponding to single pixels identified. Scale bar = 20 μm. (**B**) The profiles of activity for the identified pixel in response to visual stimuli. Spikes with the peak exceeding a z-score of 5 are indicated in red. (**C**) Trace details of a subset ROIs during the last series of visual stimulations.

In order to extract the information regarding the neuropil area encoding the sensory input, we implemented a pixel-wise regression-based analysis^7^. Briefly, the fluorescence time series of each pixel was fit with a linear model of the expected signal elicited by the visual stimulation considering the characteristic time constant of the functional reporter. This allowed mapping the RGC axonal projections in the different AFs and the corresponding spots of activity in response to multiple presentations of a visual stimulus to the contralateral eye, as in zebrafish RGC project exclusively to the contralateral brain hemisphere. The visual stimulation consisted in the sequential presentation of one, two or three small looming dots presented three times. Activity associated with the presentation of the visual stimuli was clearly detectable above the noise floor from single pixels (0.655 ± 0.1713, mean ± standard deviation) (Fig. 3).

### Whole brain neuronal recordings in zebrafish larvae

For dense recording of the neuronal activity across the whole brain, we configured the acquisition parameters to sample a volume corresponding to about 800 × 400 × 180 μm^3^ (W × L × H), including almost completely the larva brain. Such a volume, considering a typical mean cell diameter of 6 μm, can be conveniently sampled with 30 planes of 1024 × 512 pixels, corresponding to an acquisition rate of 1 volume per second (vps) or alternatively 2 vps at 512 × 256 pixels. Because of the sawtooth control signal driving the ETL, each plane is slightly tilted in the shorter direction of the frame (Y axis), with the first row on average 6 μm lower than the last row of the same frame. In the typical scenario of acquisition at 1 vps, on average, the spatial sampling is 56.2 pixels per cell, with an integration time of 3.2 μs per cell, comparable to typical values of the fast-random access multiphoton acquisitions. For optimizing the spatial sampling and facilitating the automatic segmentation of the cells, it is common to use zebrafish larvae expressing nuclear localized activity reporter GCaMP6s, e.g. line Elavl:H2B-GCaMP6s. This version of the fluorescence reporter presents a characteristic response decay time between 3.5 and 4.1 s^8^. Hence, sampling cell activity at 1 vps still allows a good reconstruction of the neuronal activity profiles^9^.

**Figure 4:**
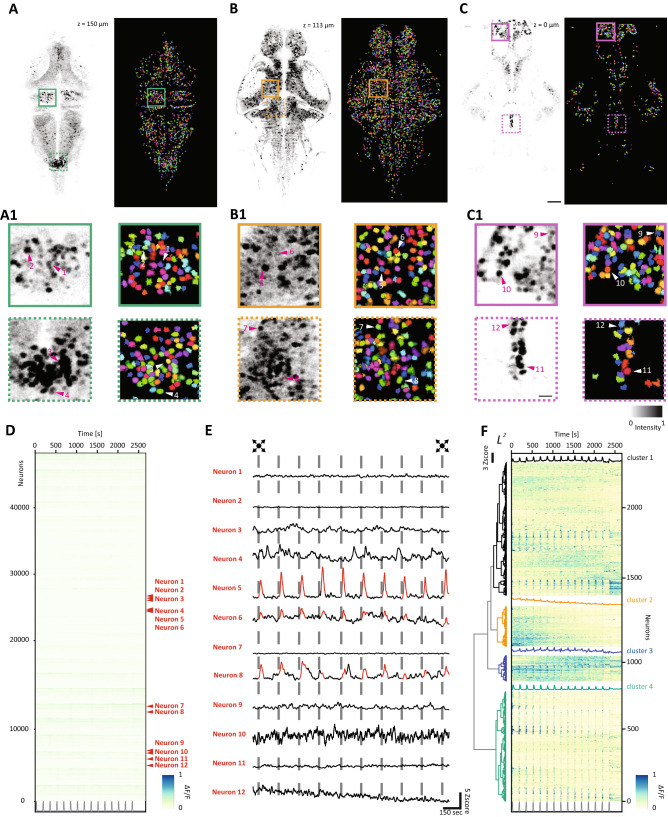
Whole brain calcium imaging. (**A–C**) Average intensity projections (left) and the results of the cell segmentation process (right) for a dorsal (**A**), central (**B**) and ventral (**C**) planes of the fish corresponding to the z-levels at + 150 μm, + 113 μm and + 0 μm, respectively. The colored boxes indicate the areas of the inset (**A1**–**C1**). Scale bar = 50 μm. (**A1–C1**) Representative fields of view of the average intensity projections (left) and of the segmentation (right) extracted from (**A**–**C**). Arrows indicate corresponding cell bodies. Scale bar = 10 μm. (**D**) Raster plot showing the activity profiles of the complete dataset of 47,992 neurons segmented as function of the time. The neurons corresponding to the (**A1**–**C1**) insets are indicated in red. The grey trace at the bottom indicates the stimulation. (**E**) The activity profiles expressed as z-score for the neurons highlighted in (**D**). The vertical bars indicate the presentation of the looming stimulation. (**F**) Hierarchical clustering and raster heatmap of the 2400 stimulus-responding neurons identified with the regression analysis. For each cluster the average response is overlaid in the corresponding color. The grey trace at the bottom indicates the stimulation.

We applied our configuration to map the brain activity on non-anesthetized 5 dpf gel-embedded zebrafish larvae presented with a monocular visual stimulation. The imaging protocol consisted of a baseline with 5 min of spontaneous activity recording followed by 30 min of sensory-evoked activity. A series of quick looming dots were presented every 150 s on a screen placed at the side of the fish. In order to obtain maps of the brain dynamics at high resolution, we refined a computational pipeline for the semi-automatic segmentation and identification of the activity profiles of single neurons (Fig. 4A–C). This procedure was mostly based on the open platform “suite2p”^27^ and relied on an automatic cell classifier trained with datasets obtained using Elavl:H2B-GCaMP6s larvae (see “Methods” section for details). Briefly, following a plane-by-plane motion correction, de-interleaved data corresponding to individual planes were filtered with a short-window running average kernel and then processed for the automatic cell segmentation routine to identify region of interest (ROI) corresponding to the individual neurons. This routine, depending on the acquisition Signal to Noise Ratio (SNR), typically led to the automatic identification of 30,000–50,000 putative cells. After two steps of manual ROI curation, for excluding false positive hits and ROIs with merged neurons, and for manually including cells not automatically detected, the typical size of the dataset resulted in 45,000–52,000 cells identified per fish (Fig. 4, Supplementary Figs. S3, S4).

From the complete activity dataset, we were able to extract a subset of 2400 neurons whose activity profile increased in response to the presentation of the stimuli (Fig. 4F). For this, we used again a generalized linear regression model to fit the activity profile of each segmented neuron with a regressor, corresponding to the presentation of the visual stimulus. For each ROI, we calculated a score (regressor coefficient/mean squared error), to identify the neurons with prominent responses to the visual stimulation. We considered a neuron to be responding to the stimulation if presenting a score within the top 5% of the complete distribution of the scores, across all the identified cells and trials (Supplementary Fig. S5). On the neuronal activity profiles of this dataset, a hierarchical clustering analysis revealed the presence of four main groups (black, orange, blue and bluish green sub-trees), with distinct profiles of response to repeated visual stimulation (Fig. 4F, Supplementary Fig. S6).

**Figure 5:**
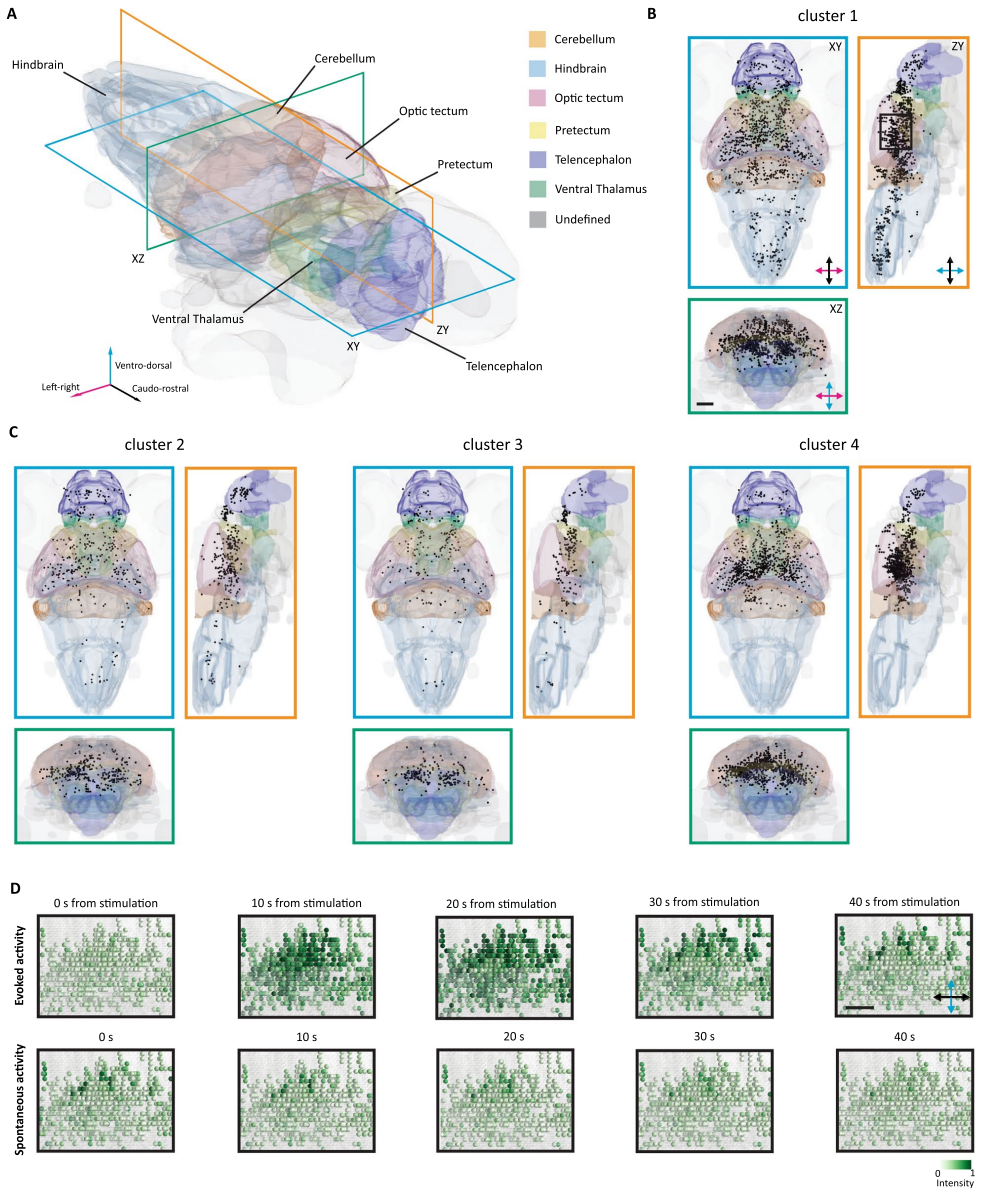
Anatomical representation of the identified clusters of visually responsive cells. (**A**) Three-dimensional representation of the brain of the larva with some of the most prominent region indicated. Three sectioning planes are overlaid to indicate the corresponding viewports for the following orthoviews. (**B**) Horizontal (cerulean), sagittal (orange) and coronal (bluish-green) views of the brain, with the projection of the stimulus-responsive cells belonging to the cluster 1. The black box indicates the area of the inset in D. Scale bar = 100 μm. (**C**) Horizontal, sagittal and coronal views of the brain, with the projection of the stimulus-responsive cells belonging to the cluster 2, 3 and 4. (**D**) Field of view extracted from the longitudinal plane in (**B**). In the top row, evoked activity of visually responsive cells is reported for 40 s from the onset of the stimulus. In the bottom row, spontaneous activity of the same cells is reported for 40 s. Scale bar = 50 μm.

Then, we mapped these functionally identified clusters on the corresponding zebrafish larvae brain (Fig. 5). Stimulus-responsive cells were identified, as expected, in the optic tectum contralateral to the stimulated eye, in regions in proximity of some of the deeper arborization fields, in the pretectum, in the ventro-lateral region corresponding to the isthmic populations, and in a few regions of the fish hindbrain (Fig. 5B,C). These maps revealed that, even though the clusters show a certain degree of overlap, some regions do present a pronounced enrichment for one of the clusters identified. This is particularly true for the cluster 4 highly represented in the optic tectum and in areas located close to the deeper arborization fields of the contralateral side of the brain. As for cluster 2, this mainly populates the midbrain region corresponding to the optic tectum. While, for the cluster 1 the map shows a high density of cells at the level of the tectum opticum and in the ipsilateral hindbrain regions. Finally, cells belonging to cluster 3 are mainly localized ventrally in areas of the pretectum and thalamus.

## Discussion

Zebrafish larvae are one of the few model organisms that allow mapping the neuronal activity at cellular resolution across most of its brain. Traditionally, this takes advantage from the combination of fluorescence-based activity reporters expressed in genetically modified zebrafish lines, with microscopy techniques relying on the extended illumination of the brain and the detection of the signal with a parallel detection. Among the different approaches developed, single plane illumination or lightsheet microscopy has become a powerful and versatile tool to reconstruct the fish neuronal dynamics brain wide^6^. This technique is based on the illumination of the sample from the side with one or more thin sheets of light that are sequentially scanned along the dorso-ventral axis of the sample. The volumetric information is reconstructed with a CMOS or CCD camera by means of a second objective that is placed normally with respect to the illumination sheet and continuously refocused at the illuminated plane. This approach typically leads to the reconstruction of the brain activity with cellular resolution at 1 or 2 volumes per second^28^. Despite these advantages, this kind of application currently requires the design of a dedicated optical layout for light sheet and a tight synchronization between the different hardware and software components. Here, we present an alternative design for 3D whole brain functional recordings at cellular resolution. Our design relies on a multiphoton laser scanning microscope integrating a resonant galvo x/y-scanner and an ETL-based remote z-focusing. From the hardware point of view, this represents a smart solution that can easily retrofit as addon in many lab imaging systems with off-the shelf optical components. The module operates by means of a simple and cost-effective solution to control the ETL in accordance with frame acquisition. With respect to digital-scanned light sheet microscopy (DLSM) approaches typically adopted for reconstructing brain activity, where the sheet of light is generated by means of the rapid scanning of a line, there are a few points worth consideration. First, the effective budget of integrated fluorescence photons. In multiphoton beam scanning approaches, like the one we present here, the effective excitation dwell time per cell is typically ranging from a few to tens of microseconds. In DLSM, where these values are typically higher and the excitation is continuous and non-pulsed in most of the cases, the number of collected photons is greater^29^. On the other side, the proportion of collected ballistic photons tends to decrease with depth within the tissue, resulting in DLSM images captured by a CMOS with relatively higher level of background noise, and consequently lower SNR. Second, inertia-free control. Even though DLSM implementation with ETLs have been reported, most of the current designs for volumetric imaging still include a piezo actuator to control either the detection or the excitation arm^30,31^, potentially introducing undesired perturbation to the experiment. We adopted a design for the remote control of the imaging plane based on an electrically tunable lens. Of all the possible reported approaches^2,11–15^, these devices can offer a good travelling range, in the order of 200–250 μm, with sufficient temporal performances, 6–12 ms settling times, at the cost of limited hardware modifications. Third, constraints associated with the illumination scheme. In DLSM microscopy, either adopting continuous wave visible or pulsed infrared excitation sources, it is common to arrange the light scanning parameters in order to avoid the light beams hitting the region of the eyes of the fish. Prolonged exposure of these regions could potentially lead to stimulation-induced visual artifacts or, in the worst cases, to alterations of the organ functionality. This typically restricts the effective imaged volume and increases the complexity of the optical layout. With respect to DLSM, our design, sequentially scanning a single excitation spot, concentrate the excitation power in a relatively smaller volume^29^. This facilitates heat dissipation and relaxes the constraints due to the maximal usable light dose^32^.

In conclusion, we presented here an alternative design for high resolution brain imaging in zebrafish larvae integrating the current available toolbox of the suitable techniques. We demonstrated that this approach can be used to reconstruct the brain dynamics during sensory stimulation. With the help of a refined analysis pipeline, we obtained datasets with 45,000–52,000 cells per fish, sufficient for an in-depth characterization of the brain mechanisms.

## Methods

### Transgenic lines and experimental preparation

Experiments were carried out according to the current legislation of our country (Decreto Legislativo 4 Marzo 2014, n.26) and were approved by the Ethical Committee of the University of Padua (61/2020_dal Maschio) and adhere to the ARRIVE (Animal Research: Reporting of In Vivo Experiments) guidelines. Larvae were raised at 28 °C on a 12 h light/12 h dark cycle using standard procedures. Ath5-GCaMP6s larvae were used for tectal neuropil imaging, Elavl3:H2B-GCaMP6s larvae were used for whole brain imaging. Transgenic lines were kept in Tupfel long fin nacre (TLN) background, carrying a mutation suppressing the melanophore pigmentation. Five days post-fertilization (dpf) larvae were embedded in 2% agarose gel and used for experiments.

### Hardware for two-photon calcium imaging

The imaging path is based on an 8-kHz galvo-resonant commercial 2P design (Bergamo I Series, Thorlabs, Newton, NJ, United States) seeded by a Ti:Sapphire source (Chameleon Ultra II, Coherent). For imaging GCaMP6, the source is tuned at 920 nm and a water dipping Nikon CFI75 LWD 16X W objective is used for all the acquisitions. The fluorescence collection path includes a 705 nm long-pass main dichroic, an IR blocking filter and a 495 nm long-pass dichroic mirrors transmitting the fluorescence light toward a high sensitivity GaAsP PMT detector (H7422PA-40, Hamamatsu) equipped with EM525/50 emission filter. The PMT signal is sampled at 400 MHz by a fast digitizer board (Alazar Tech Board) and the images, acquired at 30 frames per second, stored in the PC in a .raw file format. The commercial system includes a control unit (MCU) which provides the analog waveforms driving the galvo-resonant scanner and other TTL synchronization signals. In particular, a 30 Hz TTL signal, named *Frame Sync*, marks the beginning of the scanning along the slow axis (Y), i.e., the beginning of the image acquisition and is used to synchronize the ETL scanning.

### Implementation and control of the ETL-based remote focusing system

An electrically tunable lens (ETL, Optotune EL-10-30-C-IR) is placed upstream to the x/y scanner, which is in a plane optically conjugated with the back focal plane (BFP) of the objective. Current is fed to the ETL by the ETL driver (Optotune Lens Driver 4i), which can be controlled either by a PC via USB with the proprietary software or by external analog and digital signals. We implemented this last strategy, controlling the ETL by generating and sending serial commands to the ETL driver. To this purpose, we added a commercially available microcontroller (Arduino Mega 2560) and connected it to the ETL driver via the UART interface.

The wiring is performed as follows. The pin 47 of the Arduino Mega 2560 corresponds to the counter input and it is connected (together with the ground (GND)) to the” Frame Sync” TTL (0-5 V) output of the Microscope Control Unit via a coaxial cable. The pins 18 and 19 are, respectively, TX and RX of the UART serial port 1; they are connected to the corresponding RX/TX pins of the ETL driver UART serial port, as described in the ETL driver manual.

The microcontroller accepts as input the frame clock signal from the MCU. This signal, *Frame Sync*, marks the beginning of the scanning along the slow axis (Y) and is used to increment a digital counter implemented in the microcontroller. The microcontroller repeatedly sends to the ETL driver, at the maximum possible rate (every 3 ms in our case), the commands for updating the value of the current *I(t)*. The value of the current *I(t)* is computed from a 5th order polynomial which interpolates the points of a LUT obtained during a calibration phase. The calibration procedure envisages the acquisition of a series of images of the same field of view, every time with different currents driving the ETL. The corresponding z-shift of the objective arm to bring the FOV back in focus represents a measurement of the focal shift introduced by the ETL. This set of *z(I)* points is used to generate the LUT. First, the *z* values are remapped to z^*^ in the range 0–1. Then, the corresponding values of current I(z^*^), where I(z^*^ = *0)* = *I*_*min*_ and I(z^*^ = *1)* = *I*_*max*_, are fitted with a 5th order polynomial, whose coefficients *p*_0_,…,*p*_5_ are then loaded in the microcontroller. This is programmed to continuously generate a sawtooth waveform, z^*^(t) (where *t* is an internal high-resolution timer), spanning the range 0–1 in the desired number of counted frames, 30 in our case. The corresponding currents I(z^*^(t)) are computed from the coefficients *p*_0,_…,*p*_5_ and commands are sent to the ETL driver to update the output current. The microcontroller, moreover, periodically sends the total number of counted frames to the PC, which is used for synchronization of the stimulation protocol. Arduino code for controlling the ETL and Python script to compute 5th order polynomial coefficients are presented in the GitHub repository.

### Optical performance measurement

To calculate the PSF, we acquired, mechanically moving the objective with respect to the sample, a z-stacks of fluorescent polystyrene beads with a nominal diameter of 0.2 μm at eight different positions set with the remote focus in the z-range between 0 and 233 μm. The z-stacks were analyzed with the Fiji distribution of the ImageJ software (https://imagej.net/Fiji/Downloads). After an initial “Re-slicing” along the given axis (either x/y or z), slices underwent a z-projection in order to obtain a slice with average intensity. Fluorescence of the bead was estimated by extracting the value obtained through the ‘Plot Profile’ function of the software. The FWHM was calculated as:

$$FWHM=2\sqrt{2\mathrm{ln}2}*\sigma$$

where $\sigma$ is the standard deviation of the beads fluorescent profile.

The FOV dimensions were measured by annotating the μm travel of the piezo actuator to shift a fluorescent bead from one side of the FOV to the other along the given axis.

### Visual stimulation

Looming stimuli were generated in Python using Stytra^33^. Stimuli were presented monocularly at 60 Hz on a 50 × 50 mm screen using a DPL4500 Texas Instrument projector placed 20 cm from the screen and equipped with a magenta Wratter filter to avoid light interference with the fluorescence detection. The screen was 3 cm from the subjects. The beginning of the generation of the stimulation sequence is triggered at a time based on the microscope frame count, thus aligning the presentation of the visual stimuli with the imaging acquisition. For the RGC arborization fields imaging, three looming dots expanded in 2 s from a diameter of 0 mm to 5 mm. The size to speed ratio (*|l/v|*) was 1 s, the angle size at the end of the stimulation was 9.5° and the angular expansion rate was 4.7°/s. The three dots were projected centered with the fish eye (dot #1), 13.75 mm caudally the fish (dot #2), 13.75 mm rostrally the fish (dot #3). The experiment consisted of 3 repetitions of the sequence: dot #1—pause—dots #1,2—pause—dots #1,2,3—pause. For whole brain imaging, a single looming dot, projected centered with the fish, expanded from a diameter of 0 mm to 50 mm in 17 s. The *|l/v|* was 8.3 s, the angle size at the end of the stimulation was 79.4° and the angular expansion rate was 9.5°/s. The stimulus was presented 10 times with 150 s intervals between the stimuli.

### Imaging data processing

Time series were recorded at one volume per seconds with a spatial resolution of 512 × 512 pixels for imaging the tectal neuropil imaging or 1024 × 512 pixels for whole brain acquisitions. In order to avoid the focus shift due to the thermal drift of the lens, the first minutes of acquisition were discarded. Raw data with the interleaved planes were first motion corrected plane by plane to minimize the effect of motion and smoothed with a short window running average filter (3 frames). For the RGC recordings, the noise floor was estimated as the mean value of the standard deviation of 16 pre-stimulus segments.

For whole brain recordings, the filtered data were then processed for automatic cell segmentation with suite2p, using a cell classifier trained on similar datasets. At the end of the automatic segmentation procedure, datasets were manually inspected to check the quality and the accuracy of the ROI classification. Typically, we found cells not properly identified as ROIs by the automatic segmentation and ROIs not corresponding to an identifiable cell. These hits were manually curated to distill a clean dataset, typically resulting in 45,000–52,000 neurons correctly identified.

### Regressor analysis

To identify pixels of the neuropil or neurons active during the presentation of the visual stimulation, we used a regression-based analysis using Python libraries. We designed an orthogonal basis of regressors corresponding to the different presentations of the visual stimulation, resulting in binary waveforms (i.e., they are most of the time at a zero-level baseline, with transitions to a high level corresponding to the frame of presentation of the stimulus baseline and return to the baseline at the end of the stimulation). To describe the impulsive response of the activity reporter, we considered the kinetics of the GCaMP sensor by convolving the regressors with a normalized exponentially-decaying kernel. The activity of single pixel or of the identified cells were either correlated with the regressor gathering all the stimulation or fitted with a linear regression model based on the different regressors by using the “Linear Regression” class of the scikit-learn library^34^. The resulting coefficients were divided for the mean squared error of the fit to obtain a set of scores. The cells, whose score was within the top 5%, or 10%, of the distribution, were further analyzed to identify functional clusters and to generate the dendrogram. The analysis was performed via the “AgglomerativeClustering” class of the scikit-learn library^34^. We used Ward’s method of linkage which is based on Lebesgue 2-norm (*L*^2^ norm).

### Anatomical registration

We acquired a z-stack of the functionally imaged fish at a resolution of 1024 × 512 pixels with a z spacing of 1 µm. The z-stack data was mapped to the averaged time series of the functionally images planes with a custom python script. The z-stack data was registered on a live standard brain^35^ using the Elav3:H2B-GCaMP6s reference channel by the “antsRegistration” command in ANTs^36^. This step generates a “0GenericAffine.mat” file and a “1InverseWarp.nii.gz” file necessary to the “antsApplyTransformToPoints” command to convert the positional information about the ROIs to the anatomical space of the standard brain. For the anatomical registration within the brain regions, the coordinates were mapped on the fixed standard brain.

### 3D rendering

By means of a custom-written Python script based on MayaVi^37^, this information was used as a reference to plot the single cells in their anatomical positions and then to visualize the rendering of the entire dataset. The meshes of the regions were downloaded from https://fishatlas.neuro.mpg.de.

### Images

Plots were generated using the ‘matplotlib’ library for Python^38^. All the images were edited using Adobe Illustrator CS2 (https://adobe.com/products/illustrator).

## Supplementary Information

Supplementary Information 1.

Supplementary Video 1.

## Data Availability

Raw imaging data are available upon request addressed to the corresponding author.

## Code availability

Arduino code for controlling the ETL and Python code to perform the analysis are available on GitHub (https://github.com/dalMaschio-lab/Bruzzone2021_tunableLens).
