# Supplementary material for: Whole brain functional recordings at cellular resolution in zebrafish larvae with 3D scanning multiphoton microscopy
Source: Sci Rep. 2021 May 26;11:11048. doi: 10.1038/s41598-021-90335-y (PMC8154985; doi:10.1038/s41598-021-90335-y)

Supplementary Information for:
"Whole brain functional recordings at cellular resolution in zebrafish larvae with 3D scanning multiphoton microscopy"

Bruzzone M.[2], Chiarello E.[2], Albanesi M.[2], Miletto Petrazzini ME.[1], Megighian A.[1,2], Lodovichi C.[1,2,3,4] and dal Maschio M.[1,2,]*

[1,]Department of Biomedical Sciences, University of Padua, via U. Bassi 58, Padova, Italy ;

[2,]Padua Neuroscience Center - PNC, University of Padua, via Orus 2B, Padova , Italy;

[3,]Veneto Institute of Molecular Medicine, VIMM, via Orus 2, Padova, Italy;

[4,]Institute of Neuroscience, CNR-IN, Padova, Italy

[,]corresponding author: marco.dalmaschio@unipd.it*

Supplementary Video

Supplementary Video S1. Cells activity during stimulation. *Animation of the 2,400 cells reported in Fig.5 during the presentation of visual stimuli.*

Supplementary Figures

Supplementary Figure S1. ETL lookup table*. Focal shift at the level of the sample as a function of the changes of the input current to the ETL. The relationship is used by the python code in Supplementary Method S1 to calculate the coefficients for the polynomial fitting (line in red).*

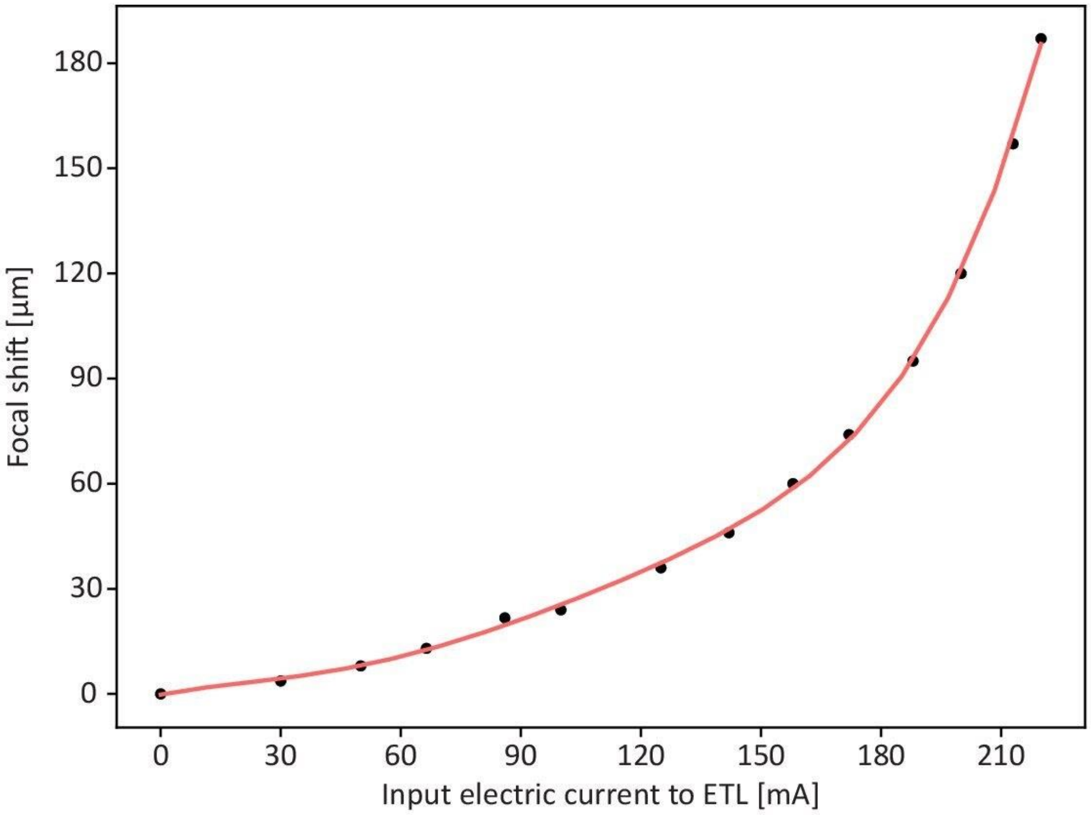

Supplementary Figure S2. FOV size. *FOV dimensions as a function of the ETL defocus and of the corresponding ETL driving current.*

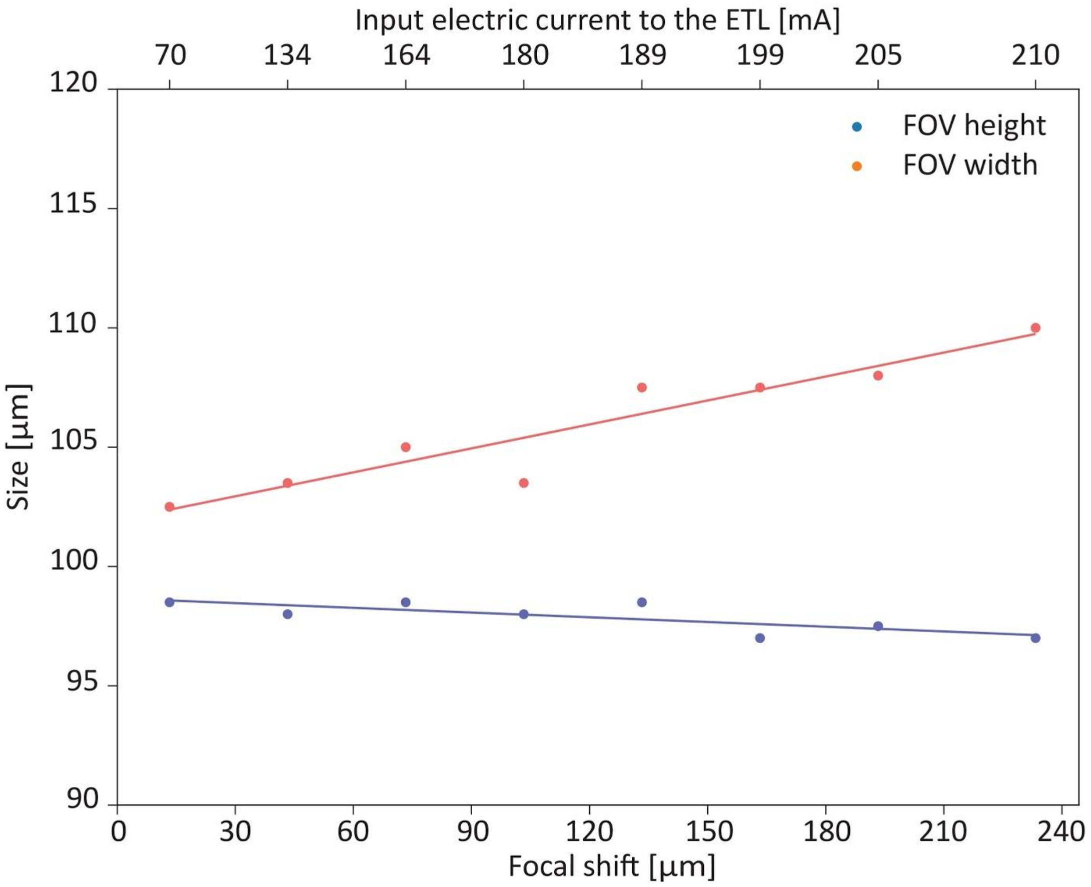

Supplementary Figure S3. Multiplane imaging of the zebrafish brain I. A. *Average intensity projections of 30 planes, encompassing almost the entire volume of the zebrafish larvae, sampled during an acquisition. Scale bar = 100 µm.* ***B.*** *The cell segmentation process of the planes led to the identification of 47,992 cells.*

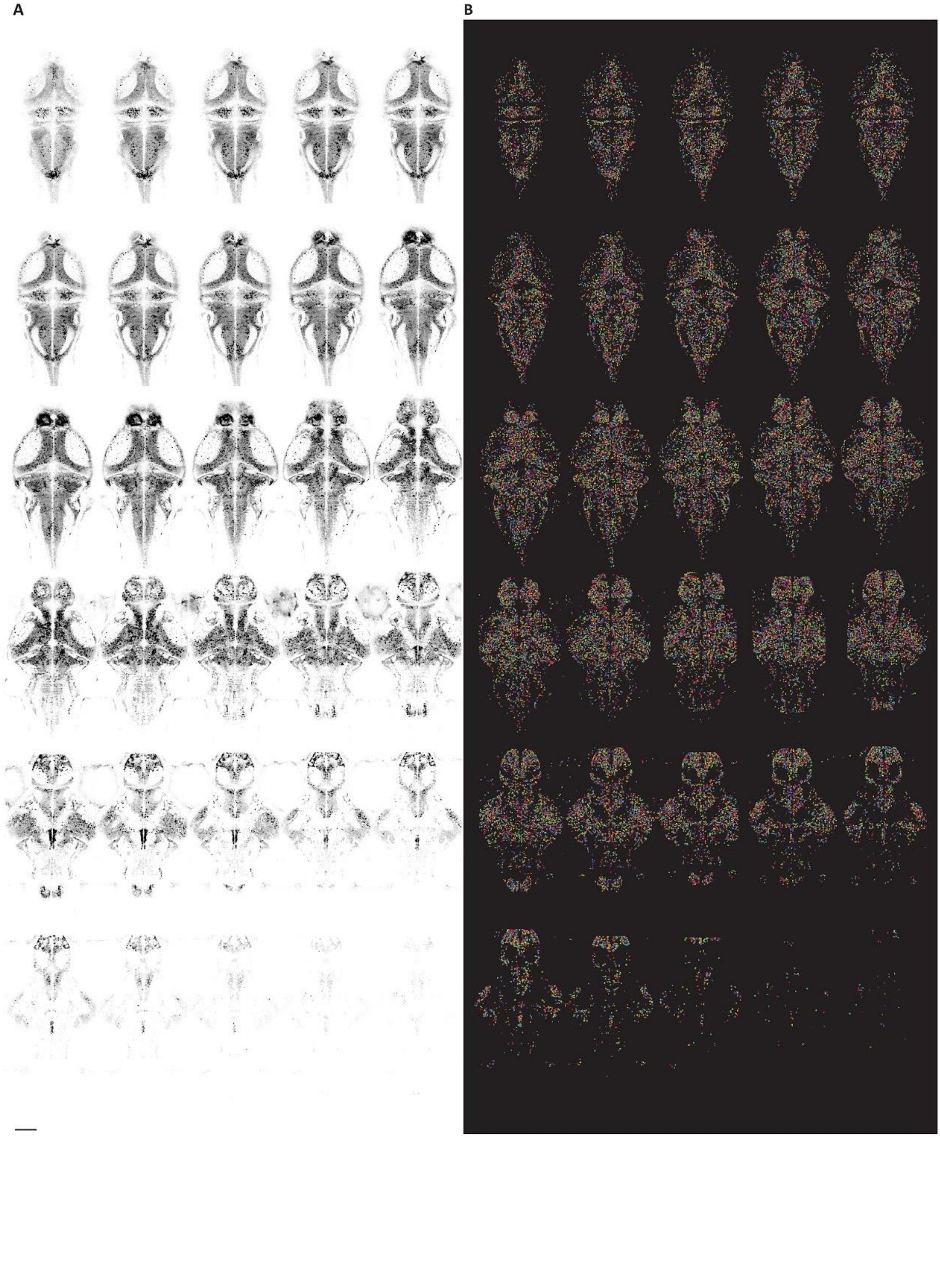

Supplementary Figure S4. Multiplane imaging of the zebrafish brain II. A. *Average intensity projections of 30 planes, encompassing almost the entire volume of the zebrafish larvae, sampled during an acquisition. Scale bar = 100 µm.* ***B.*** *The cell segmentation process of the planes led to the identification of 52,949 cells.*

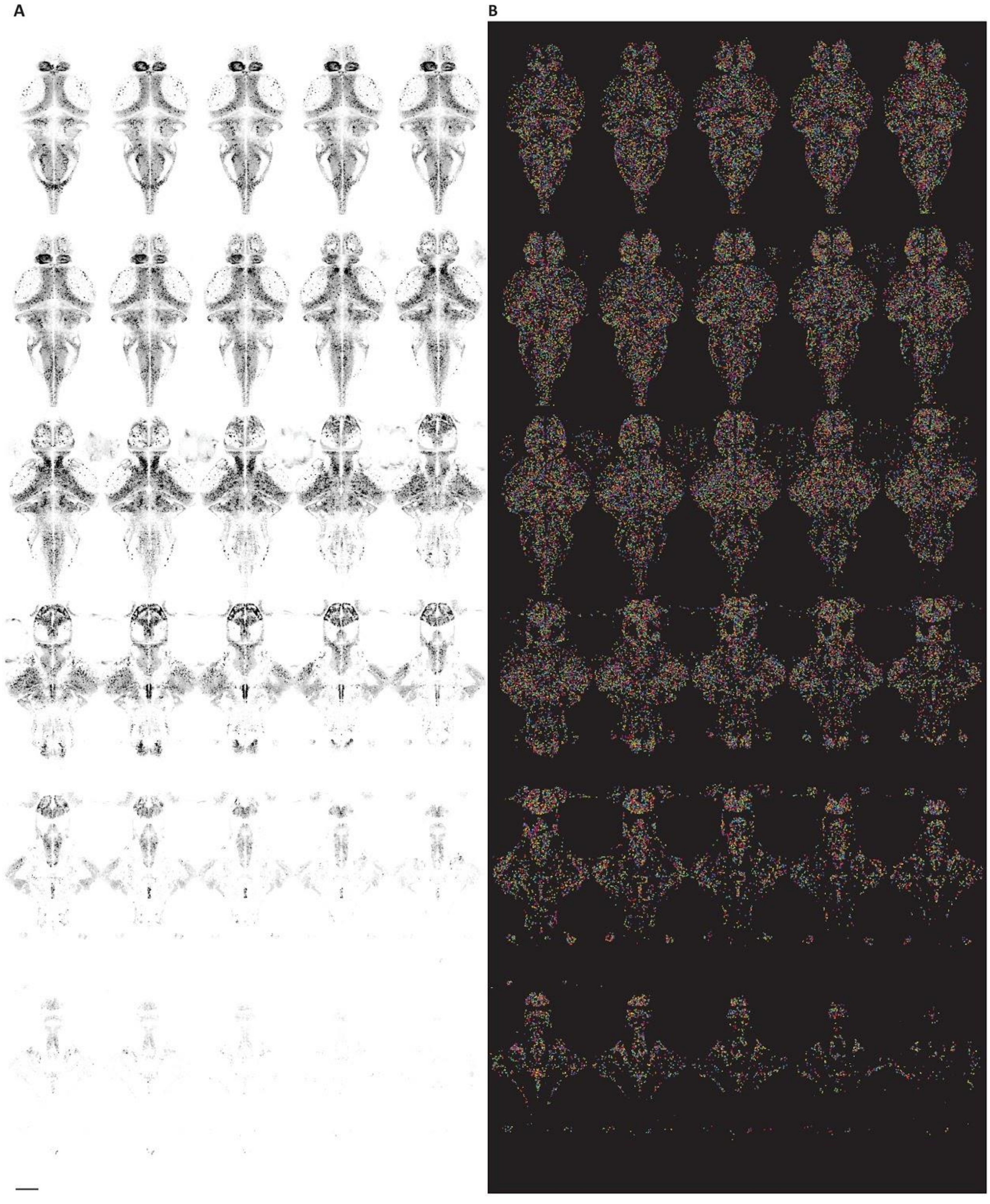

Supplementary Figure S5. Correlation coefficients. A-B. *Distribution of the correlation coefficients for the whole-brain imaging dataset with highlighted in dark the 90th and 95th percentile, respectively.*

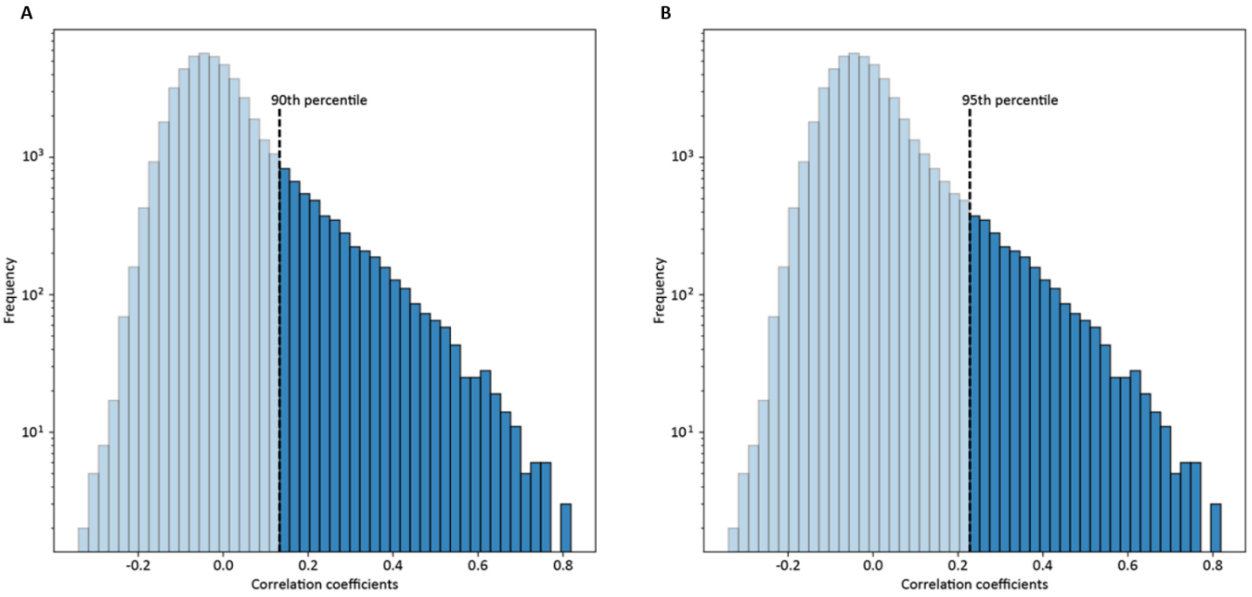

Supplementary Figure S6. Identified clusters of visually responsive cells with a 10% threshold. A. *Hierarchical clustering of the 4,800 stimulus-responding neurons identified with the regression analysis. For each cluster the average response is overlaid in the corresponding color. The grey trace at the bottom indicates the stimulation.* ***B.*** *Horizontal (cerulean), sagittal (orange) and coronal (bluish green) views of the brain, with the projection of the stimulus-responsive cells belonging to the clusters identified in panel.*

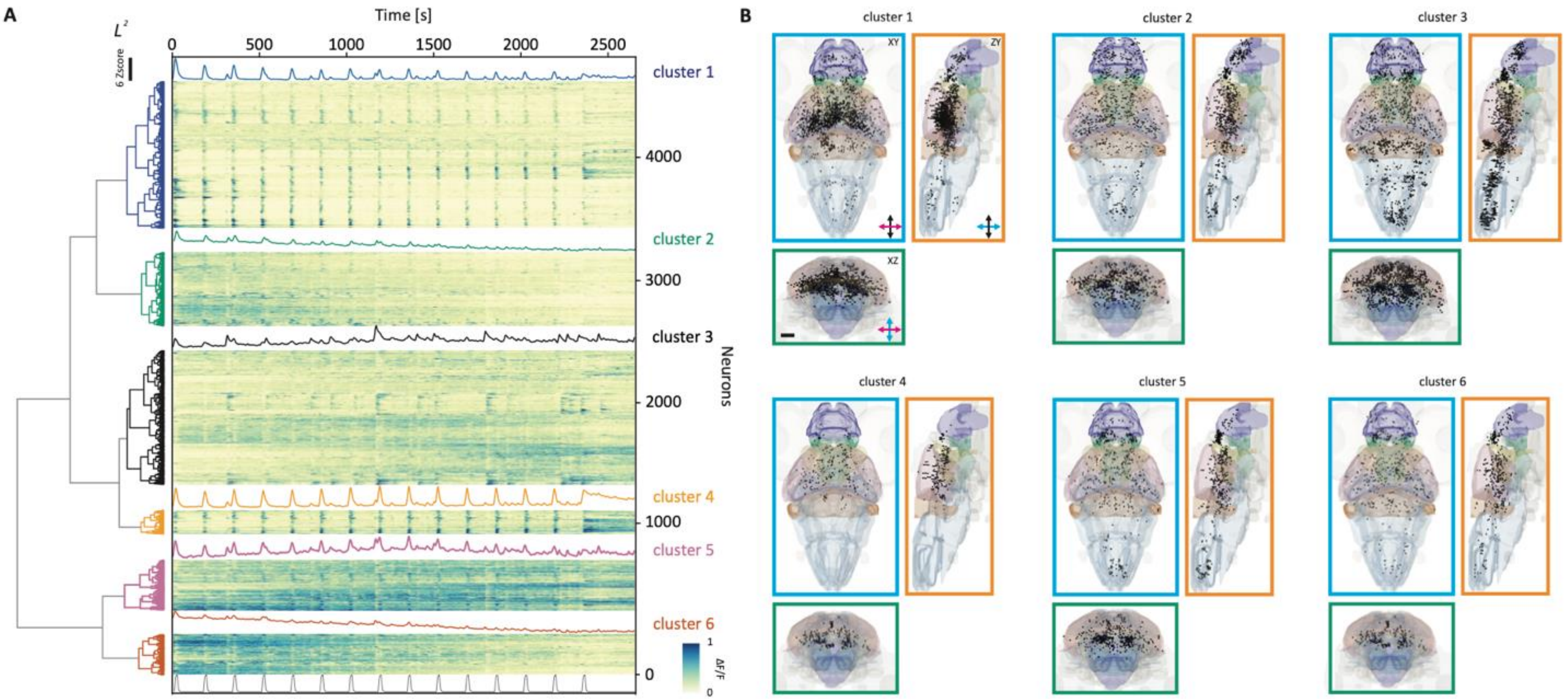

Supplement: Supplementary file 1 — Supplementary Information 1. [file 41598_2021_90335_MOESM1_ESM.pdf]
